# Assessment of microcirculation variables and endothelial glycocalyx using sidestream dark field videomicroscopy in anesthetized dogs undergoing cardiopulmonary bypass

**DOI:** 10.3389/fvets.2023.1189738

**Published:** 2023-08-21

**Authors:** Devon M. Diaz, E. Christopher Orton, Marlis L. de Rezende, Kristin Zersen, Julien Guillaumin

**Affiliations:** Department of Clinical Sciences, College of Veterinary Medicine and Biomedical Sciences, Colorado State University, Fort Collins, CO, United States

**Keywords:** dogs, cardiac disease, tricuspid valve, extracorporeal circulation, endothelium

## Abstract

**Introduction:**

To evaluate microcirculation and endothelial glycocalyx (eGC) variables using sidestream darkfield (SDF) videomicroscopy in canine cardiopulmonary bypass (CPB).

**Methods:**

Dogs undergoing CPB for surgical correction of naturally-occurring cardiac disease were prospectively included. Variables collected included patient demographics, underlying cardiac disease, red blood cell flow (Flow), 4-25 μm vessel density (Density), absolute capillary blood volume (CBVabs), relative capillary blood volume (CBVrel) and eGC width assessed by perfused boundary region (PBR). Anesthetized healthy dogs were used as control. Microcirculation and eGC variables were compared at baseline under anesthesia (T0), on CPB prior to cross clamping (T1), after cross clamp removal following surgical correction (T2) and at surgical closure (T3).

**Results:**

Twelve dogs were enrolled, including 10 with a complete dataset. Median Flow was 233.9, 79.9, 164.3, and 136.1 μm/s at T0, T1, T2, and T3, respectively, (*p* = 1.00). Median Density was 173.3, 118.4, 121.0 and 155.4 mm/mm^2^ at T0, T1, T2, and T3, respectively, (*p* = 1.00). Median CBVabs decreased over time: 7.4, 6.6, 4.8 and 4.7 10^3^μm^3^ at T0, T1, T2, and T3, respectively, (*p* < 0.01). Median CBVrel increased over time: 1.1, 1.5,1.1, and 1.3 10^3^μm^3^ at T0, T1, T2, and T3, respectively, (*p* < 0.001). Median PBR increased over time: 1.8, 2.1, 2.4, 2.1 μm at T0, T1, T2, and T3, respectively, (*p* < 0.001). Compared to control dogs (*n* = 8), CPB dogs had lower CBVabs at T0.

**Conclusion:**

Alterations in eGC thickness and microvascular occur in dogs undergoing CPB for naturally-occurring cardiac disease.

## Introduction

The microcirculation is defined as the terminal vascular network of the systemic circulation consisting of micro vessels with diameters <20 μm (1). The microvascular unit is comprised of arterioles, post capillary venules, capillaries and their sub cellular components (2). In recent years, the endothelial glycocalyx (eGC) has been recognized as an important part of that microvascular unit (3). The eGC is a gel-like structure that covers the luminal surface of the vascular endothelium, and is composed of a network of glycosaminoglycans, glycoproteins, and proteoglycans with the predominant glycosaminoglycans being heparin sulfate, chondroitin sulfate, and hyaluronan (4–6). The eGC has been recognized for its pivotal role in regulating hemostasis, permeability, and inflammation (7). The microcirculation is commonly assessed at the sublingual mucosa using hand-held sidestream dark field (SDF) videomicroscopy (2, 3, 8). Recently, a novel SDF video microscope^1^ has been launched, which combines microcirculation video microscopy and measures the perfused boundary region (PBR) as a surrogate for eGC thickness (9–12). An increased in the size of the PBR is due to the dynamic lateral movement of red blood cells into the permeable part of the eGC, indicating a thinner, or damaged, eGC (11).

Changes in microcirculation parameters and eGC shedding have been demonstrated during cardiopulmonary bypass (CPB), a procedure commonly performed in people, but seldomly in clinical veterinary medicine (13–16). Using SDF microscopy and eGC shedding biomarkers such as syndecan-1, heparan sulfate, and hyaluronan, it has been shown that CPB and cardiac surgery in people are associated with decreased vessel density and flow index, and increased PBR (17–20). No association with outcome have been identified. There is no published data regarding microcirculatory changes assessed by SDF, or eGC shedding in experimental or clinical CPB in dogs.

The goal of our study was to investigate changes in microcirculatory parameters using SDF microscopy and assess PBR in client-owned dogs undergoing CPB for correction of a naturally occurring cardiac defect. We hypothesize that dogs undergoing CPB will have changes in microcirculatory parameters and an increased PBR.

## Materials and methods

Dogs that presented to the James L. Voss Veterinary Teaching Hospital at Colorado State University from November 2020 to June 2022 were prospectively enrolled in the study if their naturally occurring cardiac disease required surgical correction under CPB. The study had prior ethical approval by IACUC and the Clinical Review Board (KP IACUC 1342). Informed written consent was obtained from the owner or legal custodian of all dogs described in this study for all procedure(s) performed.

### Anesthesia and monitoring

At the discretion of the board-certified anesthesiologist, dogs were premedicated with a pure μ opioid [i.e., hydromorphone (0.1–0.2 mg/kg), methadone (0.6–0.8 mg/kg) or morphine (1 mg/kg); subcutaneous or intramuscular]. Some dogs also received atropine (0.02 mg/kg subcutaneous or intramuscular) or glycopyrrolate (0.01 mg/kg intramuscular) as clinically indicated and at the anesthesiologist’s preference to maintain heart rate above 60 beats per minute. Anesthesia was induced with fentanyl [5–12 μg/kg Intravenous (IV), midazolam (0.2–0.3 mg/kg IV) and etomidate as needed (0.1–0.3 mg/kg IV) to achieve endotracheal intubation. In two cases where sedation was considered poor, propofol (1 mg/kg IV) or alfaxalone (0.25 mg/kg IV) were administered to augment sedation and facilitate induction of anesthesia. General anesthesia was maintained with isoflurane, fentanyl (10–40 μg/kg/h IV) constant rate infusion (CRI)], midazolam (0.2–0.3 mg/kg/h IV CRI), and lidocaine (30–120 μg/kg/min IV CRI). Blood pressure support was provided with dopamine (0.5–7 μg/kg/min IV CRI), dobutamine (1–10 μg/kg/min IV CRI) and/or phenylephrine (0.5–1 μg/kg/min IV CRI) as clinically indicated to maintain a mean arterial pressure above 60 mmHg prior to going onto CPB. Cefazolin (22 mg/kg IV) was administered every 90 min during surgery. Positive pressure ventilation was maintained throughout the procedure except during full CPB. During this period, isoflurane was administered via vaporizer placed in the gas line supplying the membrane oxygenator (19).

A 7-Fr triple lumen catheter^2^ was placed percutaneously into a jugular vein to provide central venous access. A dorsal pedal arterial catheter was placed to monitor direct arterial pressure and for arterial blood gas analysis, including lactate. Electrocardiogram, arterial blood pressure, central venous pressure, end tidal CO_2_, pulse oximetry and esophageal temperature were monitored continuously during anesthesia. Arterial blood gasses, acid–base status and electrolytes, activated clotting time,^3^ hematocrit and total protein were measured periodically throughout the procedure (19).

### Cardiopulmonary bypass procedure

Dogs were positioned in lateral recumbency for CPB and surgery. Prior to cannulation for CPB, heparin sulfate (300 units/kg, IV) was administered to achieve complete anticoagulation, defined as an activated clotting time over 480 s (14). Antifibrinolytic therapy with epsilon amino-caproic acid (50 mg/kg IV over 30 min) was administered as a bolus before initiation of CPB and maintained as a constant rate infusion (10 mg/kg/h) during CPB. The femoral artery was cannulated after surgical cut down with a 12 or 14 Fr low profile arterial cannula.^4^

Surgery was performed through a right (for tricuspid valve procedure) or left (for mitral valve procedure) fifth intercostal thoracotomy and pericardiotomy. For surgical repairs requiring a right atriotomy, tapes with Rommel tourniquets were placed around the cranial vena cava, caudal vena cava and azygous vein. Bicaval venous cannulation of the cranial and caudal vena cava was accomplished with 20 Fr and 22 Fr malleable single-stage venous cannulae,^5^ respectively. The caudal caval cannula was introduced via the dorsocaudal right atrial wall or the tip of the right auricle. The venous cannulae were connected to the venous reservoir/membrane oxygenator^6^ via a Y-piece. For surgical repairs requiring a left atriotomy, a single dual-stage 20/22 Fr venous cannula was introduced into the right atrium via the right auricle. This was defined as timepoint T0.

The primary CPB circuit consisted of a centrifugal pump, reservoir/membrane oxygenator, heat exchanger, and circulating heater/cooler water bath (21). The CPB circuit was primed with a balanced crystalloid solution. Additives to the crystalloid prime were mannitol (0.5 mg/kg), sodium bicarbonate (15 mg/L), and heparin (1,000 U/L) (21). Dogs were hemodiluted by mixing their blood volume with the circuit prime and cooled to a rectal temperature of 28°C (82.4°F). Extracorporeal blood flow was adjusted to a flow rate of 2.4 L/m^2^/min measured by a flow meter placed on the arterial line. Timepoint T1 was defined as dogs under CPB.

With the CPB pump on, the periaortic fat was dissected from the ascending aorta and a tape was passed around the ascending aorta. Two sutures were placed in the ascending aorta and passed through Rommel tourniquets. A 5 Fr cardioplegia cannula^7^ was introduced into the ascending aorta and secured.

The ascending aorta was cross-clamped distal to the cardioplegia cannula. Cold (4°C, 15 mL/kg) sanguineous cardioplegia solution was administered and repeated as appropriate during aortic cross-clamp, approximatively every 45 min (14).

Metabolic acidosis during CPB was corrected by administration of sodium bicarbonate (0.5 to 1 mEq/kg) into the CPB circuit as needed to maintain a pH between 7.35 and 7.45 (21).

After the specific surgical procedure was complete and cardiotomy closed, the aortic cross-clamp was removed, and coronary circulation was reestablished. The heart was electrically defibrillated, if necessary, with direct current (20 to 50 J), using internal paddles. Timepoint T2 was defined as the heart began beating on its own. Dogs were warmed to 38°C [100.4°F] and gradually weaned from CPB. During this period, calcium gluconate (30 mg/kg, IV) was administered to keep the pH-corrected ionized calcium above 1.2 mmol/L. Dobutamine (1 to 10 μg/kg/min, IV CRI) and phenylephrine (0.5–3 μg/kg/min IV CRI) were administered as necessary to maintain a mean arterial pressure above 60 mmHg.

During weaning from CPB, the left heart was vented by suction via the cardioplegia cannula. After weaning from CPB, at the discretion of the primary surgeon (CO), arterial–venous modified ultrafiltration was performed for 15–20 min with an ultrafiltration device^8^ to remove excess water.

Once the dog had been weaned from CPB and was hemodynamically stable, cannulae were removed in the reverse order that they were introduced, and protamine sulfate (1.0 to 3.0 mg/kg, IV CRI) was administered slowly (over 30 min) until the activated clotting time returned below 120 s. Thoracotomy was closed in routine fashion, and defined T3.

### Surgical procedures

Surgical procedures included tricuspid valve repair (14), artificial chordae or edge-to-edge mitral valve repair with annuloplasty (15, 16), ventricular septal defect repair with aortic valve repair (22), and partial atrioventricular septal defect repair (22).

### Data collected

Demographic variables recorded included sex, breed, age, body weight and underlying cardiac disease. Macrocirculation variables reported were heart rate (HR), invasive mean blood pressure (MBP) and serum Lactate. Microcirculation variables were assessed using a SDF video microscope with proprietary software to calculate PBR as a surrogate for eGC thickness. All variables were recorded at the following timepoints: T0 (baseline), defined as the patient stable for at least 5 min under general anesthesia; T1 (on pump), defined as a patient on CPB prior to the aortic cross clamp being applied; T2 (after clamp), defined as after the aortic cross clamp was removed after the surgical procedure was performed, while still on CPB; and T3 (closure), during surgical closure when the patient was weaned off CPB.

### Microcirculation variables

At the timepoints defined previously, a SDF video microscope was placed on the sublingual mucosa (see text footnote 1). The video microscope uses a 540 nm green light emitting stroboscopic diode to detect hemoglobin of passing red blood cells. Using a 5x objective with 0.2 numerical aperture, images were captured, providing a 325-fold magnification in 720 × 576 pixels.

The software records movies of 1 s that consist of 23 frames. Recording is initiated automatically when the software deems the images of sufficient quality, meaning that the intensity and focus are sufficient for calculations and that the camera is held sufficiently still. Vessels are automatically detected, and measurement points are defined at 10 μm intervals. The software limits its calculations to vessels with a width between 5 and 25 μm. A measurement is complete when 3,000 measurement points have been acquired.

Two researchers (DD and JG), both experienced in performing sublingual measurements with this device, performed one video acquisition for each dog at each timepoint. Care was taken to limit pressure artifacts by ensuring erythrocytes could be seen traveling through the blood vessels during the measurements. Images were recorded only in the absence of air bubbles, excessive amounts of saliva, excessive loops of the vessels and excessive amounts of large venules. All measurements in each dog at each timepoint were performed within 5 min.

The analysis of all images was performed using a proprietary software.^9^ The following microcirculation variables were recorded at each timepoint. Red blood cell flow (Flow) was defined as the velocity of a red blood cell within the individual vessel. It was determined by dividing the displacement of the RBC by the time between video frames and expressed as μm/s (11). Vessel density (Density) was determined by multiplying the number of vascular segments containing RBCs by the capillary segment length. The software detected vessels that contain more than 50% RBCs with a velocity greater than zero. Densities of vessels from 4 to 25 μm were analyzed (11). The absolute capillary blood volume (CBVabs) was determined from the number of capillary segments multiplied by the capillary segment length and the segment specific capillary cross sectional area (11). Capillary (5–7 μm) blood volume relative (CBVrel) to larger vessels (10–25 μm) was a functional estimate using the absolute capillary blood volume and comparing the average volume of red blood cells in capillaries to larger blood vessels (11).

The PBR was calculated by the software as the dynamic lateral movement of red blood cells into the permeable part of the eGC. The inner layer of the glycocalyx is penetrable to red blood cells and, hence, also called PBR (23). An intact glycocalyx is thicker and less penetrable to red blood cells, leading to a thinner PBR, compared to a damaged glycocalyx. The PBR is thus an inverse measure of the glycocalyx thickness. The proprietary software calculates the PBR from the intensity profile at every measurement point (23). A more gradual increase of the intensity profile means a thicker PBR – indicating a thinner glycocalyx (11).

### Control group

As no data has been published using this specific SDF video microscope, microcirculatory variables and PBR measurements were performed for normal dogs under general anesthesia. (KP IACUC 2237). In brief, 8 healthy purpose-bred and ethically sourced Beagles were sedated with hydromorphone (0.1 mg/kg IM), induced with propofol (5–10 mg/kg IV to effect), and intubated. General anesthesia was maintained with isoflurane. Once stabilized under general anesthesia with an invasive mean arterial pressure of 70–80 mmHg for 10 min, the SDF video microscope was placed on the sublingual mucosa. Images were captured and the analysis of all images was carried out with a proprietary software to acquire the aforementioned variables.

### Statistical analysis

Descriptive statistics were used to describe patient demographics, underlying cardiac disease and surgical procedure performed. Because of the small sample size, data were reported as median (range) and non-parametric tests were used. Impact of the CPB and surgical procedures on HR, MBP, Lactate, Flow, Density, CBVabs, CBVrel and PBR between each time point was tested with Friedman test for repeated measures. Differences between specific timepoints in the CPB group were tested with a Wilcoxon test for paired samples. At T0, each measured variables (i.e., HR, MBP, Lactate, Flow, Density, CBVabs, CBVrel and PBR) was compared to the control group using an unpaired Mann–Whitney test. Significance was set at *p* ≤ 0.05. All statistics were performed using commercially available software.^10^

## Results

A total of 13 dogs were prospectively enrolled. A device malfunction did not allow appropriate recording for all time points in one dog. Data were not recorded on T1 for 1 dog due to device malfunction, and on T4 for one dog due to cardiopulmonary arrest with unsuccessful cardiopulmonary resuscitation. Therefore, 12 dogs were included in the study, and 10 dogs had complete data sets available to be analyzed using Friedman test for repeated measures.

### Demographics

In the CPB group (*n* = 12), dogs had a median age of 1.8 years (0.7–6.9) and a median body weight of 29.6 kg (12.4–54.0). There were 7 females (4 spayed) and 5 males (1 neutered) included. Mixed breed dogs were the most frequently represented dog breed (*n* = 3). Other breeds included: Labrador Retrievers (*n* = 2), Australian Shepherd (*n* = 2), German Shepherd (*n* = 2), and one of each of the following breeds: Doberman, Rottweiler, and Great Dane.

Underlying cardiac disease diagnoses included tricuspid valve dysplasia (*n* = 5), degenerative mitral valve disease (*n* = 3), Tetralogy of Fallot (*n* = 1), peri membranous ventricular septal defect (*n* = 1), mitral valve dysplasia (*n* = 1), and partial atrioventricular septal defect (*n* = 1). Surgeries performed under cardiopulmonary bypass included: tricuspid valve repair (*n* = 5), Alferri Stich of the mitral valve and annuloplasty (*n* = 2), ventriculotomy with patch graft (*n* = 1), ventricular septal defect repair with eight coronary cusps repair (*n* = 1), mitral valve repair with aortic root tear repair (*n* = 1), mitral valve repair and annuloplasty (*n* = 1) and partial atrioventricular septal defect patch (*n* = 1). The macrocirculation variables showed significant changes over time for HR and Lactate, but not for MBP (Table 1).

**Table 1:** Microcirculation and macrocirculation variables, and endothelium glycocalyx assessed by perfused boundary region.

	CPB dogs T0	CPB dogs T1	CPB dogs T2	CPB dogs T3	Control
Total RBC flow (μm/s)	233.9* (162.1–2313.9)	79.9* (9.7–729.7)	164.3 (64.6–604.2)	136.1 (25.8–1121.7)	278.7 (103.3–419.1)
Total 4–25 μm Vessel Density (mm/mm^2^)	173.3 (82.9–582.7)	118.4 (31.7–238.2)	121.0 (32.8–418.2)	155.4 (41.6–344.3)	237.1 (103.5–334.3)
Capillary Blood Volume Absolute (10^3^μm^3^)	7.4^#*^ (1.5–17.9)	6.6^#^ (1.1–21.7)	4.8^#^ (1.6–25.5)	4.7^#^ (2.3–15.4)	18.3^*^ (4.4–21.9)
Capillary Blood Volume Relative (10^3^μm^3^)	1.1^#^ (0.7–1.6)	1.5^#^ (0.6–2.2)	1.1^#^ (0.8–2.1)	1.3^#^ (0.7–2.9)	1.26 (0.94–1.38)
Perfused Boundary Region (μm)	1.8^#^ (1.3–3.4)	2.1^#^ (1.5–2.7)	2.4^#^ (1.5–3.0)	2.1^#^ (1.1–2.9)	2.37 (1.77–2.59)
Heart Rate (beats per minute)	97.5^#^ (70–145)	105.0^#^ (65–180)	102.5^#^ (80–140)	115 ^#^ (75–140)	93 (71–153)
Mean Blood Pressure (mm Hg)	77.5 (60–110)	70 (55–90)	67.5 (20–100)	80 (60–110)	71.5 (68.0–89.0)
Serum lactate (mmol/L)	0.85^#^ (0.10–2.30)	1.25^#^ (1.10–5.20)	3.10^#^ (1.80–5.00)	2.10^#^ (2.10–4.20)	1.0 (0.8–2.1)

Dogs with naturally occurring cardiac disease corrected under cardiopulmonary bypass (only complete data sets (*n* = 10) are presented). T0, Baseline under general anesthesia; T1, On the cardiopulmonary bypass pump; T2, After the aortic cross clamp was removed; T3, Off cardiopulmonary bypass, during thoracotomy surgical Closure. Anesthetized control dogs (*n* = 8) are also presented. ^#^*p* value less than < 0.001 across the same row for CPB dogs across study time points. *p value less than 0.05 between specific values within the same row.

In the control group (*n* = 8), dogs had a median age of 5 years (3–5) and a median body weight of 8.8 kg (6.6–11.5) (*n* = 8). They were all female spayed Beagles.

### Microcirculation variables and PBR changes over time in CPB dogs

Ten dogs had a complete data set and therefore had variables analyzed over time. Median time between T0 and T1 was 91 min (30–127). Median time between the start of the CPB and T1 was 11.1 min (5.3–42.5). Median time between T0 and T2 was 206 min (158–263). Median time between T0 and T3 was 254 min (184–333).

**Figure 1:**
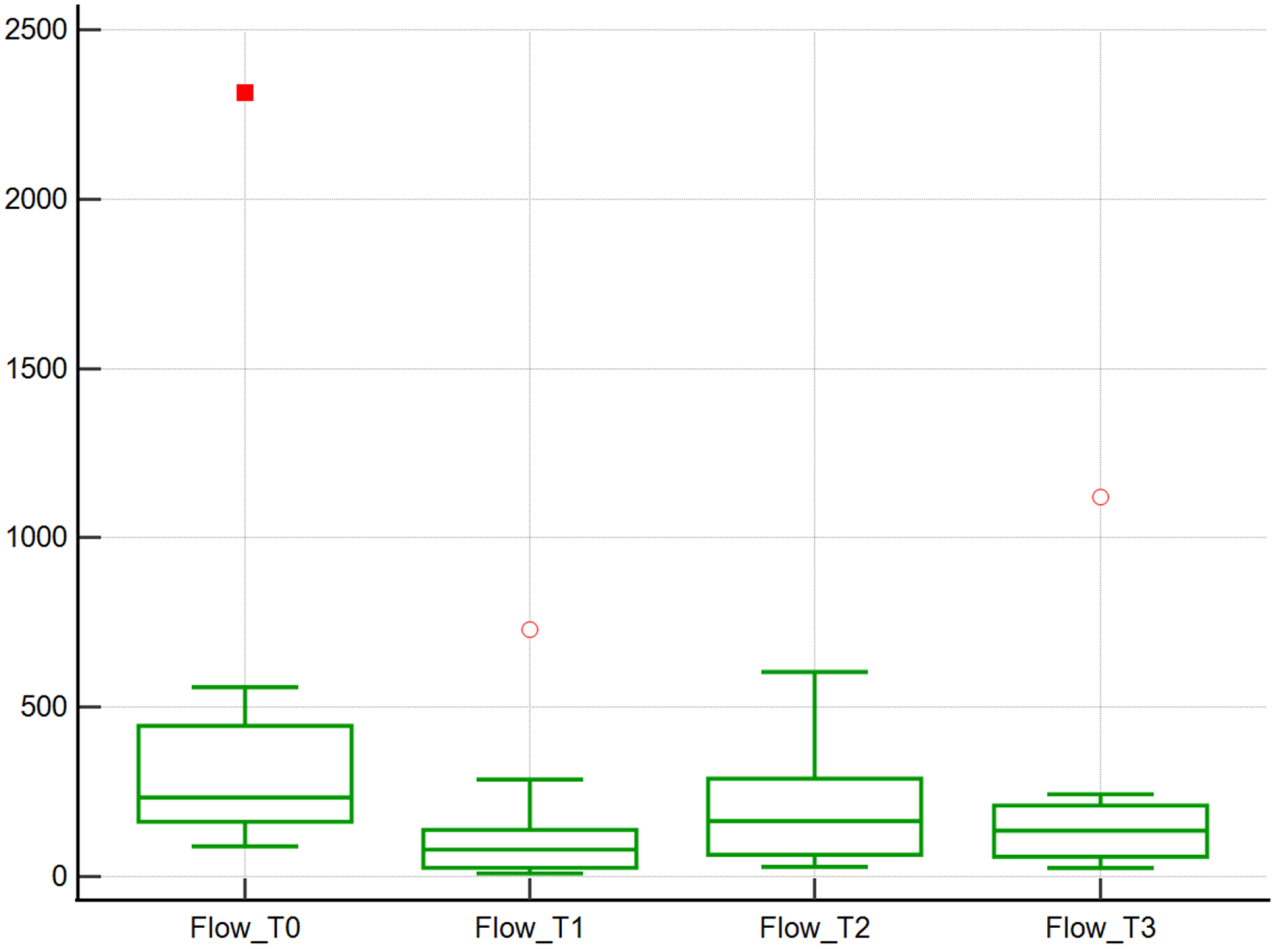
Red blood cell flow assessed using SDF. Red blood cell flow (μm/s) of 10 dogs with naturally occurring cardiac disease corrected under cardiopulmonary bypass. T0, Baseline under general anesthesia; T1, On the cardiopulmonary bypass pump; T2, After the aortic cross clamp was removed; T3, Off cardiopulmonary bypass, during thoracotomy surgical closure.

**Figure 2:**
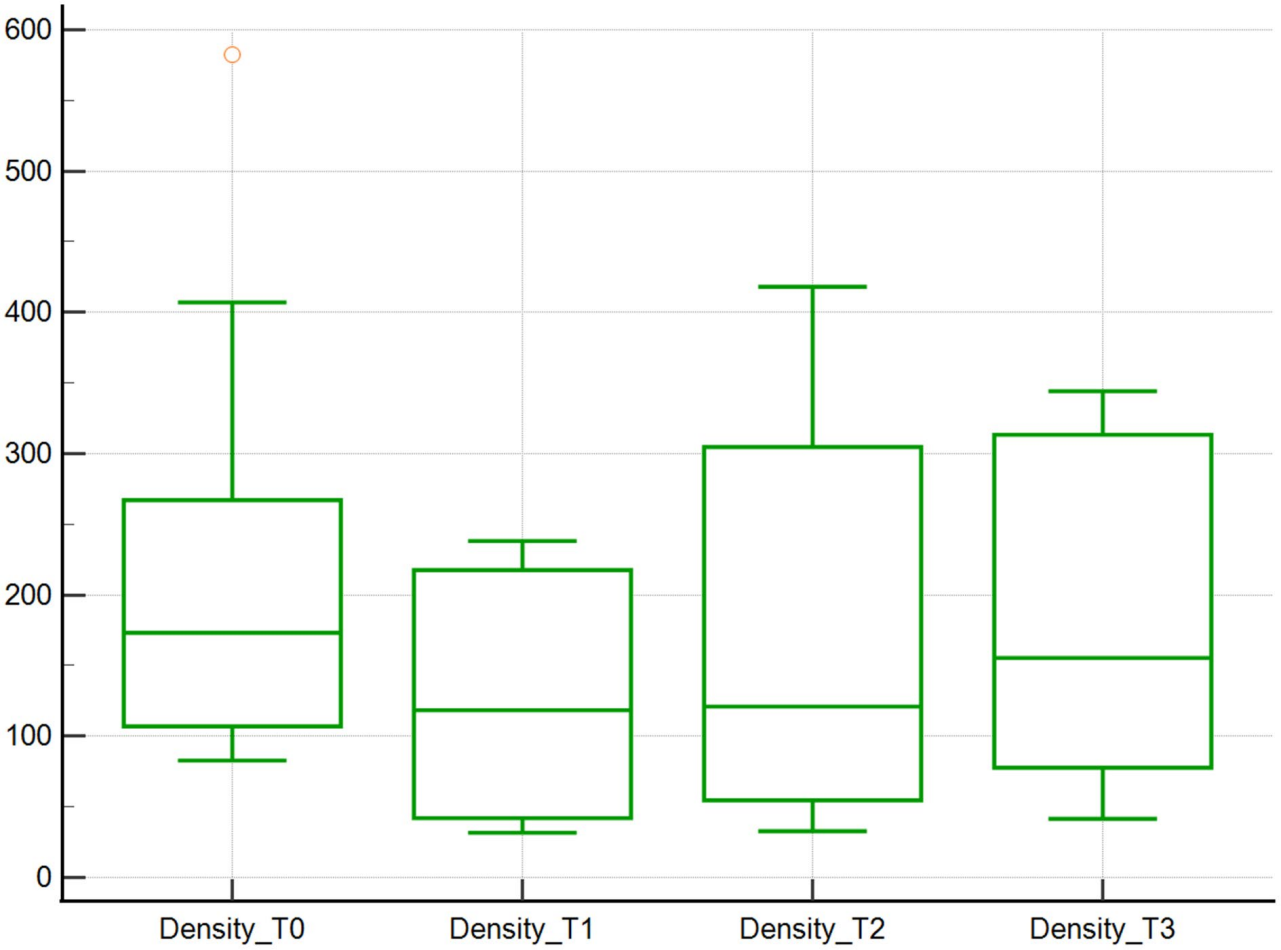
Density of vessels from 4 to 25 μm assessed using SDF. Vessel density (mm/mm^2^) of 10 dogs with naturally occurring cardiac disease corrected under cardiopulmonary bypass. T0, Baseline under general anesthesia; T1, On the cardiopulmonary bypass pump; T2, After the aortic cross clamp was removed; T3, Off cardiopulmonary bypass, during thoracotomy surgical closure.

Across the studied timepoints, no changes in the microcirculation variables Flow and Density were detected (*n* = 10). Specifically, median Flow values were 233.9 μm/s at baseline (T0), and then decreased by 65.8, 29.8 and 41.8% at T1, T2, and T3, respectively, (*p* = 1.00; Table 1; Figure 1). However, when comparing T0 and T1, the T1 Flow was significantly lower compared to baseline (T0) (*p* < 0.01). Median Density values were 173.3 mm/mm^2^ at baseline (T0) and then decreased by 31.7, 30.2 and 10.3% at T1, T2 and T3, respectively, (*p* = 1.00; Table 1; Figure 2).

**Figure 3:**
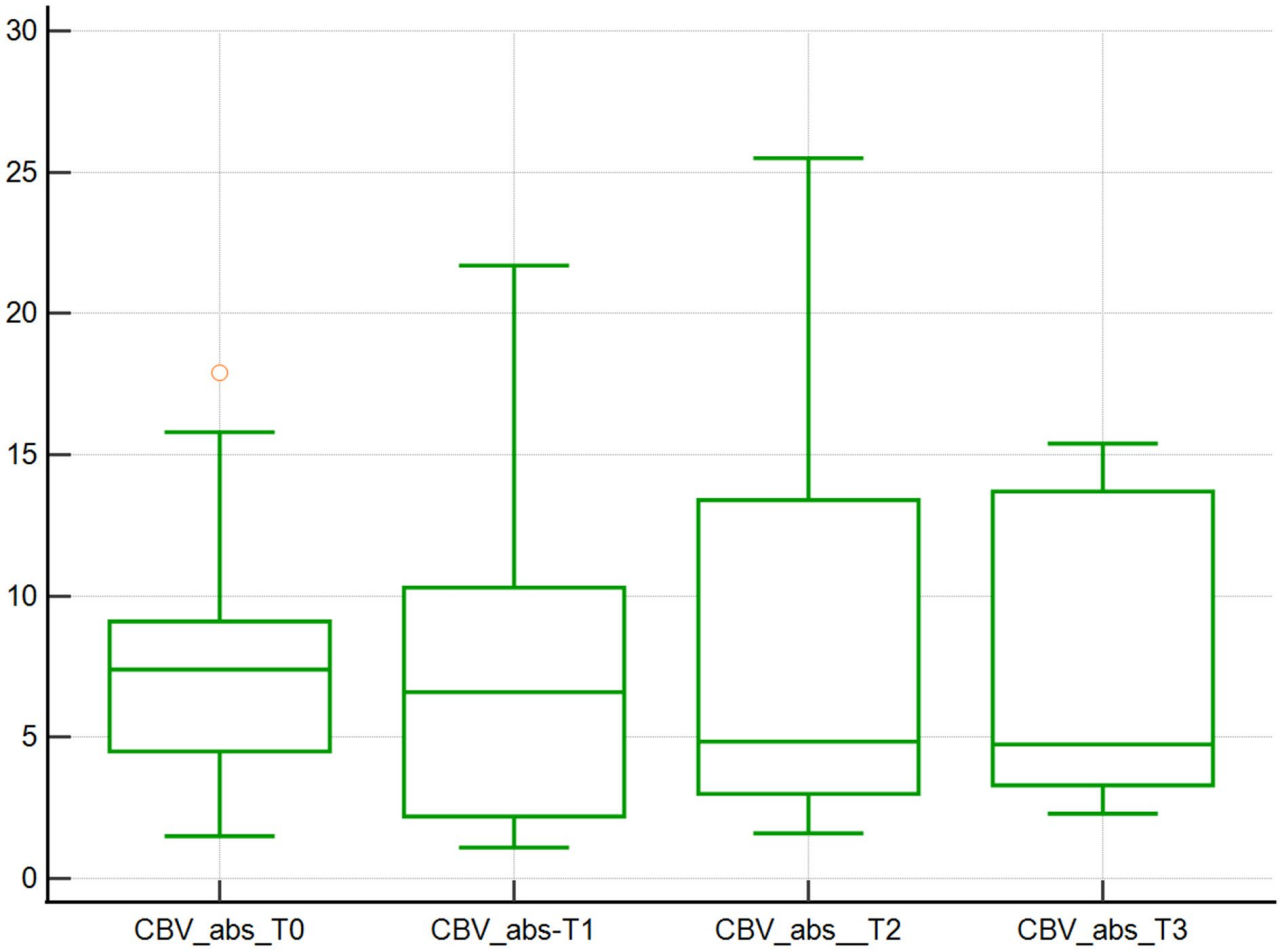
Absolute capillary blood volume assessed using SDF. Absolute capillary blood volume (10^3^μm^3^) of 10 dogs with naturally occurring cardiac disease corrected under cardiopulmonary bypass. T0, Baseline under general anesthesia; T1, On the cardiopulmonary bypass pump; T2, After the aortic cross clamp was removed; T3, Off cardiopulmonary bypass, during thoracotomy surgical closure.

**Figure 4:**
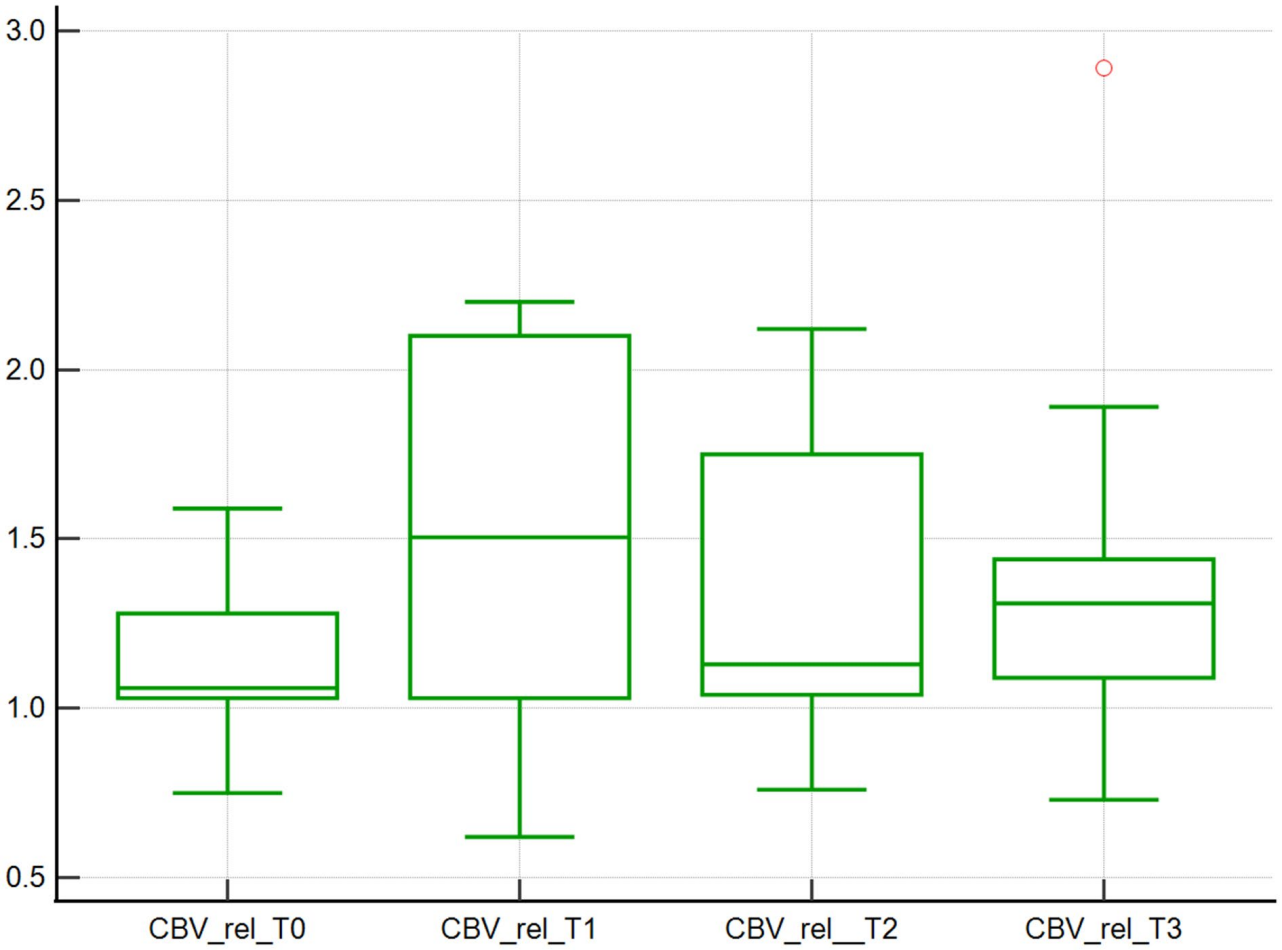
Relative capillary blood volume assessed using SDF. Relative capillary blood volume (10^3^μm^3^) of 10 dogs with naturally occurring cardiac disease corrected under cardiopulmonary bypass. T0, Baseline under general anesthesia; T1, On the cardiopulmonary bypass pump; T2, After the aortic cross clamp was removed; T3, Off cardiopulmonary bypass, during thoracotomy surgical closure.

Changes in the microcirculation variables CBVabs and CBVrel were found across the 4 time points studied. Specifically, the median CBVabs were 7.4x10^3^μm^3^ at baseline (T0), and then decreased by 10.8, 35.1, and 36.5% at T1, T2, and T3, respectively, (*p* < 0.001; Table 1; Figure 3). The median CBVrel were 1.1x10^3^μm^3^ at baseline (T0), increased by 36.4% at T1, returned to baseline at T2 (0% change), and increased by 18.2% at T3 (*p* < 0.001; Table 1; Figure 4).

**Figure 5:**
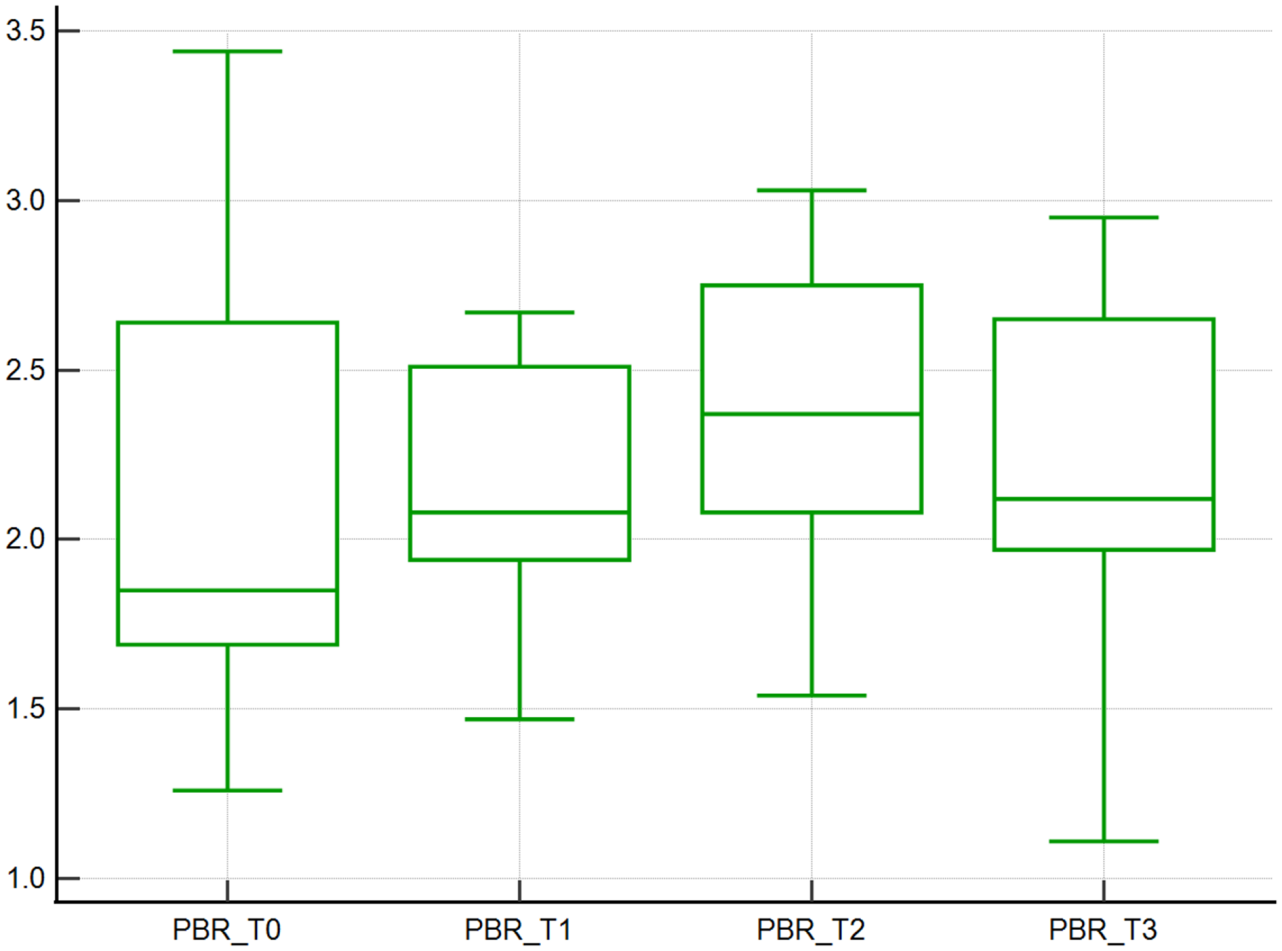
Perfused boundary region. Perfused boundary region (μm), calculated by the GlycoCheck software, of 10 dogs with naturally occurring cardiac disease corrected under cardiopulmonary bypass. T0, Baseline under general anesthesia; T1, On the cardiopulmonary bypass pump; T2, After the aortic cross clamp was removed; T3, Off cardiopulmonary bypass, during thoracotomy surgical closure.

Similarly, the median PBR increased over time, with values of 1.8 μm at baseline (T0), and increased by 16.7, 33.3, and 16.7% at T1, T2, T3, respectively, (*p* < 0.001; Table 1; Figure 5).

### Microcirculation and macrocirculation variables, and PBR in CPB dogs at T0 compared to control dogs

At T0, the microcirculation variables and PBR did not differ between the control population (*n* = 8) and CPB cases (*n* = 12) except for CBVabs. Specifically, the median Flow values were 298.7 μm/s (89.7–2313.9) and 278.7 μm/s (103.3–419.1) for the CPB and control group, respectively (*p* = 0.68). The median Density values were 173.3 mm/mm^2^ (70.4–582.7) and 237.1 mm/mm^2^ (103.5–334.3) for the CPB and control group, respectively (*p* = 0.68). The mean CBVabs were 18.3 10^3^μm^3^ (4.4–21.9) for the control group compared to 7.4 10^3^μm^3^ (1.5–18.3) for the CPB cases (*p* = 0.02). The median CBVrel were 1.08 10^3^μm^3^ (1.22–3.44) and 1.26 10^3^μm^3^ (0.94–1.38) for the CPB and control group, respectively (*p* = 0.39). The median PBR were 1.80 μm (1.22–3.44) and 2.37 μm (1.77–2.59) for the CPB and control group, respectively (*p* = 0.13).

At T0, there were no statistically significant differences between HR, MBP and Lactate between control dogs and CPB dogs (Table 1).

## Discussion

This is the first study using a SDF video microscope to measure PBR as a surrogate for eGC thickness in client-owned dogs with a clinical disease. Our study showed that PBR increased overtime in dogs undergoing CPB for surgical correction of a cardiac defect, suggesting eGC disruption. Our study also showed several changes over time in microcirculation, HR and Lactate variables, as well as a difference in CBVabs between our CPB group at T0 and our control group.

Our study showed that baseline PBR in CPB dogs is comparable to healthy, anesthetized control dogs. With a median of 1.80 μm, there were no statistically significant difference compared to a median of 2.37 μm in control dogs. The PBR in our control dogs is similar to values found in healthy anesthetized dogs and healthy sedated cats in other studies (24, 25). Our study also found that baseline PBR for dogs were comparable to studies in infants (approximately 2.5 μm), and human adults (approximately 2.0–2.2 μm) (17, 18). Our study showed a significant increase in PBR during CPB in dogs, which is consistent with most studies in people. A study in 17 adults undergoing CPB showed an average difference of 0.3 μm between baseline and after CPB weaning, as well as between baseline and 3-days post-CPB (17). Changes in PBR during CPB are confirmed by another study from the same group of investigators, where 26 adults undergoing CPB showed an increase in PBR from 2.0 um to 2.5 μm at 72 h (18). Interestingly, that study included two timepoints similar to our study, using an immediately pre-CPB, immediately post-CPB and 1-h after surgery timepoints. They showed no differences using a repeated measure ANOVA, while our study showed a difference within the timeframe of CPB (18). Similarly, in 36 infants undergoing CPB, a PBR difference was only detected at 24-h post CPB (20). It appears that changes in PBR may extend past the CPB period, and into the recovery phase in people, although our study showed a significant difference within the anesthesia period. As it is challenging to perform SDF in un-sedated or unanesthetized dogs, our study does not extend PBR measurement beyond the anesthesia period. An alternative approach to assessing the eGC would be measurements of eGC serum biomarkers such as syndecan-1, heparan sulfate or hyaluronan, which appear to change earlier and more drastically compared to PBR, and do not require sedation or general anesthesia (17, 19).

Our study found that median Flow values for dogs undergoing CPB went from a median of 233.9 μm/s at baseline (T0) down to a median of 79.9 μm/s at the start of the CPB. This can be explained by the fact that the extracorporeal blood flow of the CPB is significantly lower compared to anesthetized dogs. A normal anesthetized dog generates a cardiac output of 4.3 L/m^2^/min compared to 1.8–2.5 L/m^2^/min generated by the CPB machine (26, 27). However, the difference over time was not statistically significant. Only 10 cases were included in the repeated measure statistical analysis, so it is possible that our study is underpowered to detect a difference. Our study findings contrast with available literature in people. Unfortunately, clinical studies in people using the same SDF used in our study report a subjective microcirculatory flow index instead of an actual flow, so direct comparisons are challenging. Specifically, in a study of infants undergoing CPB, an acute reduction in flow index was seen when comparing baseline to immediately post CPB, with a drop in the microcirculatory flow index from 3.2 to 2.9 (20). It should be noted that although statistically significant, both values represent a continuous flow in their grading system. The same study also noted an inverse correlation of the microcirculatory flow index with CPB duration. However that specific datum was not presented (20).

In our study we were unable to detect a statistically significant decrease in vessel density in dogs undergoing CPB, although the lack of significance may be attributed to our small sample size, and contrasts with available literature in people which documents a progressive decrease in vessel density during CPB and a return to baseline. Specifically, in a study on 30 adults undergoing CPB and elective cardiac valve surgery evaluated using the same SDF device showed a significant decrease in vessel density from baseline to splitting of the sternum. The same study found this decrease intensified during CPB but returned to baseline in 24 h (19). The decrease in vessel density immediately after the onset of CPB was also found in another study on adults undergoing elective coronary artery bypass graft surgery although the vessel density remained altered for up to 72 h (17). Sample size, but also age of the population, may explain the lack of difference found in our study. A study in infants reported that the vessel density remained unaffected in all groups, although that specific datum is not provided (20). A direct comparison cannot be made to a study in cats due to the use of a software update (25).

The capillary blood volume variables are new features available after a software update in the Fall of 2020. This represents the physical space available for blood flow (CBVabs) as well as that space relative to the actual size of the red blood cells (CBVrel) (11). Our study found significant differences in both CBV variables across all times for CPB dogs. This can be related to changes associated with CPB and possibly changes in the flow. As there are limited data to compare to, and some of the Flow and Density changes were not significant in our study possibly due to the small sample size, further comparisons are limited.

Our study found changes in macrocirculatory parameters HR and Lactate over time. The MBP was unchanged over time, probably related to the efforts from the anesthesiologist team to maintain a normal blood pressure. Changes in HR, lactate and Flow between 2 specific time points are consistent with a hemorrhagic shock model using a different SDF video microscope (28). The discrepancy between macrocirculatory changes and microcirculatory derangements, or loss of hemodynamic coherence is very well described in the veterinary literature (3).

The variable CBVabs was significantly lower in CPB dogs at baseline (T0) compared to control dogs. In our study, the median CBVabs in healthy control dogs, measured at 18.3 10^3^μm^3^ was fairly similar to values in healthy human adults, with a median of 16.5 10^3^μm^3^ (11). Therefore, it appears that both cardiac diseases, without or without CPB induce microvascular changes highlighted by CBVabs. This may implicate altered mechanisms of compensation in dogs compared to people and warrants further investigation. Although no data exist in people undergoing a CPB procedure using this variable, that difference appear to be equivalent to that found in septic people. Specifically, septic human adults had a median CBVabs of 7.9 10^3^μm^3^, similar to the baseline values of the CPB dogs presented in this study, at 7.4 10^3^μm^3^ (11). Given no reference ranges exist prior to this study further studies are warranted to confirm the effects of underling cardiac disease on the microcirculation of dogs.

The variable CBVrel was relatively unchanged between CPB dogs and control dogs at T0. As the difference between CBVabs and CBVrel is that CBVabs investigates specifically capillary blood volume whereas CBVrel uses a comparison between capillary blood volume and larger blood vessels, those two parameters are not necessarily related. It is possible that the blood volume in capillary and larger vessels changes in unison, therefore CBVrel stays relatively unchanged. This is the first time this variable has been reported in published literature.

Although our study adds to the growing literature of eGC and microcirculatory variables in veterinary medicine and is the first to report full microcirculatory variables and eGC thickness surrogate in dogs, it is not without limitations. Most importantly, our results should be considered pilot due to our small sample size, and our study likely suffers from a type II error (29). Although our institution has a robust caseload of CPB cases, the impact of the coronavirus pandemic in 2020–2022 limited case recruitment, as has been described in people (30). The heterogeneity of cases may also hinder our ability to parse out nuances that may be attributed to the different underling cardiac disease processes, as illustrated by the wide range of values found in many variables of our study. Similarly, our control group was small, homogenous in sex, bred and age, and numerically inferior to our study dogs, which may limit data interpretation (27, 29). Finally, and due to time constraints and the dynamic nature of the procedure, only a single video was acquired at each time point, compared to three to five videos in other studies (17, 19, 20, 25). Multiple videos may have strengthened the quality of the data set.

## Conclusion

CPB induced changes in eGC thickness and microvascular changes in dogs suffering from naturally-occurring cardiac disease. The correlation between eGC and microvascular changes with clinical outcome is unknown in CPB, although there is evidence of a correlation between eGC degradation and disease severity and mortality in sepsis (31).

## Data availability statement

The raw data supporting the conclusions of this article will be made available by the authors, without undue reservation.

## Ethics statement

The animal study was reviewed and approved by IACUC committee, Colorado State University Clinical Board Review Committee, Colorado State University. Written informed consent was obtained from the owners for the participation of their animals in this study.

## Author contributions

DD: study design, data collection, manuscript draft writing, and final version editing. EO and MR: study design and final version edition. KZ: study design, data collection, and final version editing JG: study design, data collection, statistical analysis, and manuscript draft writing and final version editing. All authors contributed to the article and approved the submitted version.

## Conflict of interest

The authors declare that the research was conducted in the absence of any commercial or financial relationships that could be construed as a potential conflict of interest.

## Publisher’s note

All claims expressed in this article are solely those of the authors and do not necessarily represent those of their affiliated organizations, or those of the publisher, the editors and the reviewers. Any product that may be evaluated in this article, or claim that may be made by its manufacturer, is not guaranteed or endorsed by the publisher.

## Footnotes

^1^Glycocheck, MicroVascular Health Solutions Inc., Maastricht, The Netherlands.

^2^MILA International Inc., Florence, KY, United States.

^3^ACT Plus Medtronic, Minneapolis, MN, United States.

^4^Bio-Medicus NextGen Femoral Arterial Cannula, Medtronic, Minneapolis, MN, United States.

^5^DLP single stage venous cannula, Medtronic, Minneapolis, MN, United States.

^6^Capiox RX Oxygenator, Terumo, Ann Arbor, MI, United States.

^7^DLP Aortic Root Cannula, Medtronic, Minneapolis, MN, United States.

^8^Hemoconcetrator, Sorin Group, Arvada, CO, United States.

^9^GlycoCheck version 5.2.2.21301, Microvascular Health Solutions Inc., Salt Lake City, UT, United States.

^10^MedCalc Statistical Software version 20.026, MedCalc Software Ltd., Ostend, Belgium.

## Abbreviations

eGC: Endothelial glycocalyx
CPB: Cardiopulmonary bypass
SDF: Sidestream dark field
Density: total 4-25 μm vessel density
Flow: red blood cell flow
CBVabs: absolute capillary blood volume
CBVrel: relative capillary blood volume
PBR: Perfused Boundary Region
IV: Intravenous
CRI: Constant Rate Infusion
